# Substance use identification and follow-up rates among commercial and Medicare health insurance members in primary care and other settings

**DOI:** 10.1186/s12875-020-01286-8

**Published:** 2020-11-01

**Authors:** John G. Baker, David R. Doxbeck, Melanie E. Washington, Angela Horton, Adam Dunning

**Affiliations:** 1 Department of Community Health and Health Behavior, School of Public Health and Health Professions, and Departments of Orthopaedics and Nuclear Medicine, Jacobs School of Medicine and Biomedical Sciences, University at Buffalo, SUNY, 3435 Main Street, Buffalo, NY 14214 USA; 2 BlueCross BlueShield of Western New York, 257 West Genesee Street, NY 14202 Buffalo, USA

**Keywords:** Substance use, Behavioral health, Primary care, Social determinants, SBIRT

## Abstract

**Background:**

The objective of this study was to investigate factors associated with substance use disorder identification and follow-up rates among samples of members of a private health insurance plan.

**Methods:**

In an observational study, samples of claims data for 2017 for Commercial and Medicare members from a private health insurer were accessed and analyzed using descriptive statistics, decision tree analysis, and linear regression models.

**Results:**

Commercial and Medicare members differed in age. Medicare members had higher rates of inclusion in a measure of substance use disorder than Commercial members, lower rates of initial short term follow-up, more opioid prescriptions from primary care provides, fewer prescriptions for opioid treatment, and higher rates of selected comorbid conditions. Mental health diagnoses and substance use disorder co-occurred frequently and to a greater extent in the Medicare sample. Among commercial members, there were primarily alcohol problems that increased with age, while opioid problems at about 10% peaked in the mid-twenties. More males were included among all substance types. The overall rate for an initial short term follow-up visit indicating initiation of treatment was 30%. There were large differences in the follow-up rates across settings with a very low rate (4.6% for alcohol and 6.9% for opioid) in primary care settings.

**Conclusions:**

These results suggest that increased attention in primary care to young adult males and to older adults, may help to reduce substance use disorder rates, especially alcohol use disorders.

## Background

Substance use disorder remains a major health problem. Alcohol use disorder and dependence continues to lead to chronic health problems and other adverse consequences, while addiction to opioid drugs has reached epidemic proportions. In a position paper [1], the American College of Physicians note that in 2014 there were 22.5 million people in the U.S. who needed treatment for an alcohol or drug problem, yet only 18% received treatment compared to 77% for hypertension and 73% for diabetes. Costs for hospitalizations for opioid related disorders quadrupled during the ten years ending in 2012.

Health insurers play an important role in understanding these problems and intervening with their members to manage and reduce the ill effects of substance use disorder. For example, a recent study found an association between behavioral health conditions and behavioral health medications and both initial and long term opioid prescriptions among patients with commercial health insurance (e.g., cox regression hazard ratios of 1.94 (CI: 1.91–1.96) for depression and 1.71 (CI: 1.69–1.73) for depression medication) [2]. In terms of intervention, behavioral health treatment for depression during residential substance use disorder treatment has been shown to be cost effective (incremental cost effectiveness ratio of $131 per each point improvement on the Beck Depression Inventory-II and $49 for each depression free day) [3].

Approximately 8% of persons with private health insurance were found to have substance use disorders in one study, yet in 2009 only 0.4% of private insurance spending was for these problems [4]. A recent study found that even with more insurance benefits and increased opioid problems, this spending only increased to 0.7% in 2012 [4].

The purpose of this study was to investigate demographic and other factors associated with substance use disorder and dependence among samples of members of a private health insurance plan to aid in reducing the ill effects of this health problem. Some of the research questions included whether these factors differed among Commercial and Medicare members (who differed primarily in age rather than in type of insurance coverage) and whether factors associated with early follow-up visits could be useful in identifying members for intervention.

## Methods

### Participants

Claims data for 2017 were accessed for male and female Commercial and Medicare members of all ages from a private health insurer in the Northeast USA. In this analysis, both commercial and Medicare members receive their health insurance via a private payer. However, the commercial population are those patients, or members, who receive their health insurance coverage via their employer or health care exchange (primarily via the employer). Medicare Advantage members are those who elect a private payer instead of Original Medicare offered by the U.S. federal government, primarily to those 65 and older (https://www.medicare.gov/sign-up-change-plans/types-of-medicare-health-plans/medicare-advantage-plans). Thus, these two groups differed primarily in age rather than type of insurance coverage. Both Commercial and Medicare members of this private payer as a population would be representative of residents of the region, with the one exception that Medicaid members are not included. The ethnic and racial distributions of the state in which the members reside are: 18.8% Hispanic or Latino and 81.2% not Hispanic or Latino; 71.8% White, 18.1% Black or African American, 9.0% Asian, 1.0% American Indian or Alaska Native, and 0.1% Native Hawaiian/Pacific Islander.

Data were extracted from these claims data and data sets were created for several samples of data. Members who met criteria for inclusion in a measure of initiation and engagement in treatment after an initial visit with a substance use disorder diagnosis were selected as a primary substance use disorder (SUD) sample for further study. A second larger sample of members with a substance use disorder here referred to as a substance use condition to distinguish the two samples (SUC sample) was selected using a different, less restrictive selection criteria based only on diagnosis.

### Procedures

These individual data sets were extracted using Excel spreadsheets and analyzed using the SPSS statistical analysis program. Descriptive statistics, decision tree analysis, linear regression models, and other analytic methods were used to analyze, summarize, and present the results in verbal, tabular, and graphical format. The Decision Tree procedure creates a tree-based classification model. It classifies cases into groups (the dependent variable) based on values of independent variables, using CHAID (Chi-squared Automatic Interaction Detection), CRT (Classification and Regression Trees), and QUEST (Quick, Unbiased, Efficient Statistical Tree) algorithms. This analytic procedure provides a validation tool for exploratory or confirmatory classification analysis [5].

The logistic regression procedure regresses a dichotomous dependent variable on a set of independent variables. A categorical independent variable is replaced by sets of contrast variables, each set entering and leaving the model in a single step. A stepwise logistic regression procedure with forward entry was used to determine the relative associations of the independent variables with the dichotomous dependent variable. The logistic regression coefficients were used to estimate odds ratios for each of the independent variables in the model.

Individual members were not contacted to obtain these data and only aggregate samples of members were involved in the analysis, summary, and presentation of the data. This study was granted an exemption from requiring ethics approval by a University Institutional Review Board.

## Results

### Commercial and Medicare members

Our sample of Commercial members for 2017 included 347,471 individuals compared to 43,174 members for the Medicare sample. The number of members in the primary SUD sample meeting the criteria for a substance use disorder was also larger (*n* = 3494 vs. *n* = 1152). During 2017, Medicare members showed a higher rate of visits with a substance use disorder diagnosis (2.6%) and a lower rate of early follow-up visits (16.1%) compared to Commercial members (0.9% and 31.4%, respectively). Our SUD sample of 2017 Medicare members was 42.8% male and included 9.4% who were age 65 and under compared to 49.8% male and 94.1% age 65 and under for Commercial members. Thus, these two groups (Medicare and Commercial members) represent differences primarily in age rather than differences primarily in type of insurance coverage.

Table 1 shows the proportion of all Commercial and Medicare members by age group in the entire health plan from which the SUD and SUC samples were drawn. Table 2 shows the proportion of all Commercial and Medicare members in the entire health plan over and under age 65 by gender. The proportions of urban and rural residents were about equal.

**Table 1:** Proportion of all Commercial and Medicare Members in the Entire Health Plan by Age Groups

Age Group	Commercial	Medicare
< 18	19.4%	0.0%
18–25	11.4%	0.0%
25–49	37.2%	1.1%
50–64	29.1%	5.5%
65–74	2.6%	52.7%
75+	0.3%	40.7%

**Table 2:** Proportion of all Commercial and Medicare Members in the Entire Health Plan Over and Under Age 65 by Gender

	Commercial		Medicare	
	Female	Male	Female	Male
Under 65	48.7%	48.4%	3.5%	3.1%
65 and Over	1.3%	1.6%	52.9%	40.5%

### Pharmacy claims

Using a sample of claims level data from the first quarter of 2017, the percentage of prescriptions for several classes of drugs among the SUD sample are shown below in Table 3. It is noteworthy that the frequency (percentage) of prescriptions for opioid drugs is higher for the Medicare group, while the percentage of prescriptions for medically assisted treatment (MAT) for opioid use disorders is lower for this group.

**Table 3:** Pharmacy prescription claims level data for several drug classes among commercial and medicare members

Drug Class	***Commercial***	***Medicare***
Opioid	4.2%	5.3%
MAT Opioid	2.6%	0.2%
MAT Alcohol	0.1%	0.0%
Antidepressant	13.1%	7.3%
Total	20%	16.1%
Other Drug Classes	80%	83.9%

Comparing primary care providers to other providers, the pattern was also different for Commercial and Medicare members in the SUD sample for prescriptions during the first quarter of 2017. For Commercial members, 57% of all prescriptions were from primary care providers, including 41% of opioid prescriptions and 47% of prescriptions for opioid medically assisted treatment (MAT). About half of antidepressant prescriptions were from primary care providers. For Medicare members, more opioid prescriptions were from primary care providers (61%), while the percent of opioid MAT prescriptions was similar to Commercial members (44%). Medicare antidepressant prescriptions from primary care providers were also higher (68%) compared to Commercial antidepressant prescriptions (51%).

### Comorbid conditions

Comorbid conditions among members included in the SUD sample compared to members not included in this sample were different for Commercial and Medicare members. Among Commercial members, 60% of members included in the SUD sample had a mental health diagnosis compared to 28.4% not included in this sample. Other differences among several selected comorbid conditions included: cardiovascular- 45% vs. 31%, hepatobiliary - 14% vs. 6%, and nutrition/metabolic - 64% vs. 54%. Among Medicare members these differences were even more pronounced and included different comorbid conditions for this older sample: mental health diagnosis - 72% vs. 37%, vascular - 70% vs. 46%, diabetes - 46% vs. 31%, lung - 78% vs. 48%, neurologic - 64% vs. 38%, and notably cognitive disorders - 38% vs. 17%.

### Commercial members

Since the sample of Commercial members was much larger and the number of members meeting the criteria for a substance use disorder was also larger, we focused on the commercial sample for the remaining analyses.

### Primary SUD sample diagnostic codes

Using a sample of initial claims for 2017, for Commercial members included in the SUD sample, ICD−10 diagnostic codes included 59% alcohol use disorder and dependence codes, 9.5% opioid codes, and 18% of codes for cannabis use disorder and dependence. Thus, a majority of claims in this sample were for alcohol use disorder and dependence problems.

### Primary SUD sample age groups and substance type

**Fig. 1:**
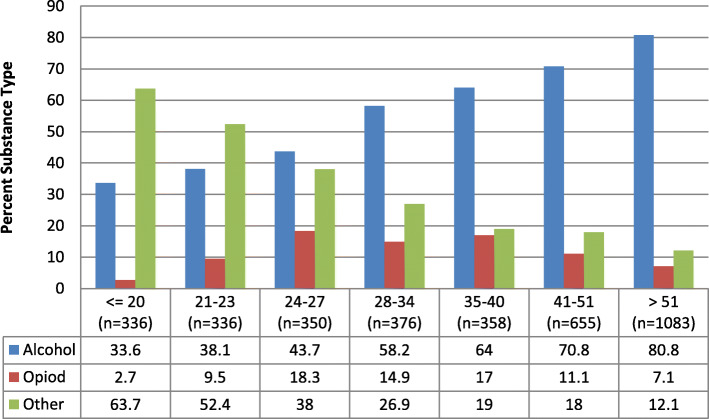
Primary SUD Sample Age Groups and Substance Type

Using age groups empirically derived from decision tree analysis, Fig. 1 shows that among Commercial members during 2017, alcohol use disorder steadily increases with age. Opioid use disorder peaks with the 24 to 27 age group and then declines, while substance use disorder of other drugs decreases with age.

### Primary SUD sample age groups and gender

**Fig. 2:**
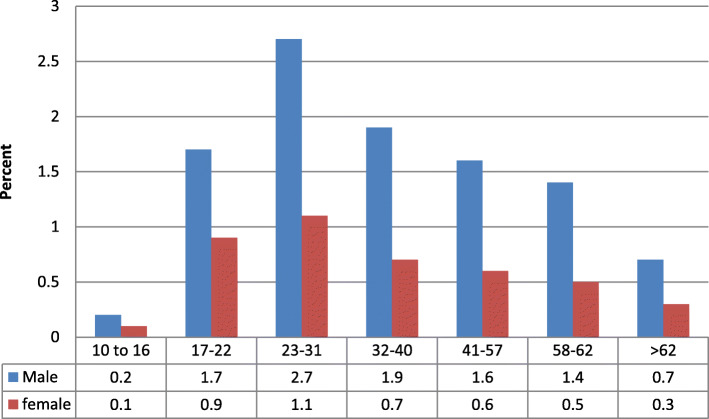
Primary SUD Sample Age Groups and Gender

Across all substances, Fig. 2 shows that more males are included in the SUD sample, and that substance use disorder peaks with the 23 to 31 year old empirically derived age group. Urban and rural member addresses were comparable across these age groups.

### Secondary substance use condition (SUC) sample

The second sample of members referred to as the substance use condition sample (SUC sample) obtained using a different, less restrictive selection criteria based only on diagnosis was considerably larger (*n* = 45,275) or 13% of the 347,454 Commercial members for 2017. This percentage is reasonably similar to the 8% rate of substance use problems found in a previous study [4].

**Fig. 3:**
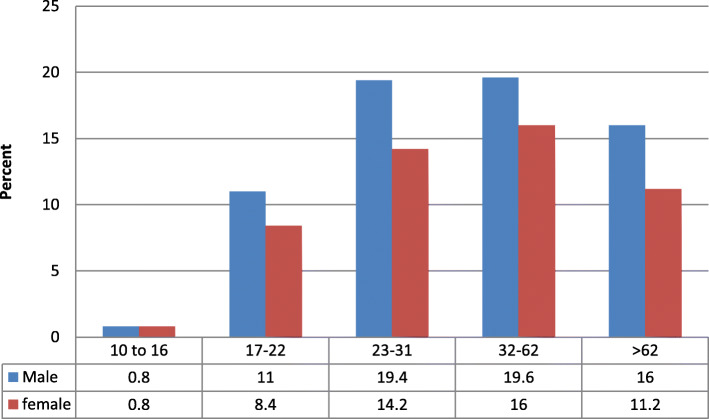
Second SUC Sample Age Groups and Gender

Figure 3 shows the percentage of Commercial members by gender included in the second larger SUC sample. The age groupings are empirically derived from decision tree analysis. More males are included and the percentage peaks with the 23 to 62 year old age group.

**Fig. 4:**
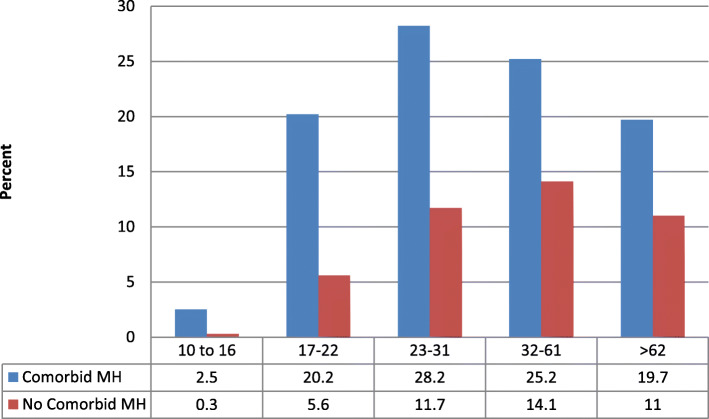
Second SUC Sample Age Groups and Mental Health Condition

Fig. 4 shows the rate of mental health conditions using empirically derived age groupings from decision tree analysis with the second SUC sample for Commercial members. The percentage of substance use disorders with a comorbid mental health condition peaks with the 23 to 31 year old age group.

### Analytic results

Stepwise logistic regression analysis was used to determine factors associated with a substance use disorder using the larger second SUC sample among Commercial members and selecting members within a more homogeneous regional market (*n* = 32,341). Several variables were associated with being included in this sample. Table 4 presents the associations and odds ratios for each of these variables.

**Table 4:** Stepwise logistic regression analysis results for inclusion/non-inclusion in the SUC sample as the dependent variable with odds ratios for each of the independent variables

Independant Variable	Beta Weight	Wald Statistic	Significance	Odds Ratio
Constant	−2.1	503.8	0.00	0.1
MH Condition	−0.8	4156.5	0.00	2.3
Age Groups		3422.4	0.00	
Age 10–16	−1.8	647.9	0.00	0.2
Age 17–22	0.9	553.9	0.00	2.4
Age 23–31	1.5	1716.6	0.00	4.4
Age 32–62	1.5	2056.0	0.00	4.5
Male-Female	−0.4	1081.5	0.00	1.5
Diabetes	−0.1	51.2	0.00	1.1
Urban-Rural	−0.1	16.6	0.00	1.1

As can be seen in Table 4 from the values of the Wald statistics (larger is more strongly associated), a mental health condition is most strongly associated with being included in the second SUC sample, then age groups, then gender, and to a much lesser extent diabetes or urban-rural member address. Based on the odds ratios, the model shows that a member with a mental health condition is 2.3 times as likely to be included in this SUC sample as a member without a mental health condition (odds ratio of 2.3). Members between the ages of 23 and 31 and 32 and 62 are 4.4 and 4.5 times, respectively, as likely to be included in this sample as members in other age groups (Odds ratios of 4.4 and 4.5). The odds ratio of 1.5 would indicate that males are 1.5 times as likely as females to be included in this larger secondary SUC sample of Commercial members.

### Primary SUD sample initial follow-up rate

Turning back again to the primary SUD sample of Commercial members that is based on the more restrictive criteria for the substance use disorder measure, a member may or may not return for an initial follow-up visit within the first 14 days. Of the 3494 Commercial members included in this measure, 1098 returned for a follow-up visit and 2396 did not (31%). As noted above, in this sample alcohol made up almost 60% of the overall substance use disorders, and the follow-up rate was 30%. For opioid use disorders the overall follow-up rate was 38%. Interestingly, the opioid follow-up rate for males (42%) was higher than for females (31%).

### Provider groups and initial follow-up

In this primary SUD sample an empirical grouping of provider specialties for opioid use disorders resulted in three groups: primary care, inpatient and program treatment, and outpatient behavioral health. The difference in follow-up percentage for specialty groups is striking, but not surprising. Inpatient or program-based treatment has a relatively higher initial follow-up rate (71.6% for opioid drugs and 59.3% for alcohol and other drugs). The follow-up percentage for primary care is very low at 6.9 and 4.6%, respectively. Although in primary care a 14 day follow-up may not be necessary to address many substance use disorder problems, nonetheless programs such as SBIRT (screening, brief intervention, and referral for treatment) may result in more patients receiving follow-up intervention in a timely manner. Follow-up in outpatient behavioral health settings is intermediate at 27.9% and 20.8%, respectively, perhaps reflecting more weekly or biweekly visits.

### Analytic results

Since the short term follow-up rate is so low for primary care providers, it would be difficult to use additional analyses to try and determine subgroups with whom to intervene. Primary care providers in general are an important group for intervention from a health insurer perspective. Among hospital and program providers, there is more of a balance between members who follow-up and those who don’t, and so additional analyses can be useful in identifying subgroups for intervention. Using stepwise logistic regression analysis with Commercial members in the primary SUD sample, several variables are associated with not following up. Table 5 presents the associations and odds ratios for each of these variables.

As can be seen in Table 5 from the values of the Wald statistics, age groups are most strongly associated with not following up, then urban-rural member address, and then substance type. Based on the Wald statistics, the model shows that ages 17 to 22 and 32 to 40 are significantly more likely to not follow up (*p* < 0.00 and *p* < 0.02, respectively). The odds ratios show that ages 17 to 22 are about 2.2 times more likely to not follow up than other age groups. An urban resident is 1.8 times as likely as a rural resident to not follow up. A member of the SUD sample using alcohol is 1.3 times as likely to not follow up compared to the other substance categories.

**Table 5:** Stepwise logistic regression analysis results for follow-up/not-follow-up in the SUD sample as the dependent variable with odds ratios for each of the independent variables

Independent Variable	Beta Weight	Wald Statistic	Significance	Odds Ratio
Constant	−0.4	5.9	0.02	0.7
Age Groups		38.7	0.00	
Age 10–16	−0.2	0.4	0.56	0.8
Age 17–22	0.8	28.1	0.00	2.2
Age 23–31	0.2	1.2	0.28	1.2
Age 32–40	0.4	5.7	0.02	1.5
Age 41–57	−0.2	1.2	0.28	0.9
Age 58–62	−0.4	3.3	0.07	0.7
Urban-Rural	−0.6	16.2	0.00	1.8
Alcohol Use Disorder	0.3	5.7	0.02	1.3
Opioid Use Disorders	−0.4	5.4	0.02	0.7

## Discussion

Several samples of claims data from a private Northeast health insurer were described and analyzed to compare Commercial and Medicare members with substance use disorders and in order to increase understanding of this population for intervention. Commercial and Medicare Members differed in age. Contrary to expectations, Medicare members had a higher rate of inclusion in a primary SUD sample based on a measure of substance use disorder than Commercial members, as well as a lower rate of initial short term follow-up. A sample of prescription claims from Medicare members showed somewhat more opioid prescriptions (5.3% versus 4.2%) and fewer prescriptions for opioid treatment (0.2% versus 2.6%) among this group. There were more opioid prescriptions from primary care provides for Medicare compared to Commercial members. The frequent co-occurrence of mental health diagnoses and substance use disorders has been noted in the literature [2], and this was seen to a greater extent in the Medicare SUD sample. Medicare members in the SUD sample also had higher rates of other selected comorbid conditions.

Commercial members in the SUD sample had mostly alcohol problems and opioid problems were about 10%. Alcohol use disorders increased with age while opioid use disorders peaked in the mid-twenties. Across all substance types more males were included in the primary SUD sample with a peak occurrence in the 23 to 31 year old age group. Using a second larger SUC sample based only on diagnosis, a mental health condition was most strongly associated with a substance use disorder.

Looking at whether Commercial members in the primary SUD sample returned for an initial short term follow-up visit as an indication of initiation of treatment, the overall rate was 30%. There were large differences in the rates across settings with a very low rate in primary care settings. Intervention with primary care providers, for example education using the Screening, Brief Intervention, and Referral for Treatment (SBIRT) model could be considered to increase the likelihood that substance use disorders are being addressed in this setting. In inpatient and program settings additional analyses found a significant association between specific age groups, urban settings, and alcohol use disorders and lack of initial short term follow-up.

There is considerable support in the literature for the integration of behavioral health services in primary care settings. The degree of integration can range from care management to the fully integrated Primary Care Behavioral Health Model [6]. A study of integration of substance use disorder treatment in primary care found that an Integrated Care Model led to significant decreases for patients with substance use disorders in hospitalization rates, inpatient days, and emergency department use, as well as a reduction in total medical costs from $431.12 to $200.03 per member month [7]. The results of the present study, including high rates of comorbid mental health diagnoses and other medical conditions among the SUD sample and the better follow-up rate for outpatient behavioral health settings, would lend additional support for a focus on primary care providers to help with improving healthcare for members with a substance use disorder.

This study has a number of limitations. Since claims data were used, the diagnosis codes for substance use disorder and other conditions were billing diagnoses rather than clinical diagnoses. For example, providers may have been reluctant to list a substance use disorder diagnosis for the purpose of billing a patient’s insurance company. Or, a substance use disorder may not have been identified by a provider. Thus, the actual rates of substance use disorder may have been higher than those reported in these claims data.

Another limitation involves the potential for sampling bias. Although the sample sizes are quite large and efforts were made not to introduce sampling bias, nonetheless a particular time period, such as the first or third quarter of a particular year may be different than other quarters or other years. The primary substance use disorder sample (SUD), which was obtained from a measure of initiation and engagement in treatment after an initial visit with a substance use disorder diagnosis, had fairly restrictive criteria for inclusion and constituted a relatively small number of members (i.e. .09% Commercial and 2.6% Medicare members). Selection for this sample may have favored some members who met these criteria over other members who did not. A second larger sample of members referred to as the substance abuse condition sample (SUC) selected using a different, less restrictive selection criteria based only on diagnosis included 13% of Commercial members. This proportion was closer to the 8% of members found to have a substance abuse problem in a previous study [4]. An important limitation is that our sample of Commercial members did not include Medicaid members and so may not be representative of this group of individuals in the region and in the state. Future research can include various samples across multiple years and investigate trends in substance use disorder rates and follow-up rates over time.

## Conclusion

Overall, these results suggest that increased attention to young adults and males in urban areas, as well as older adults, around substance use, especially alcohol, may help to reduce rates of substance use disorders. Alcohol use disorders increased with age while opioid use disorders peaked in the mid-twenties. Intervention with primary care providers may increase the likelihood that once identified, substance use disorders receive follow-up in the short term, perhaps including referral for treatment, if this is needed.

## Data Availability

The datasets generated and/or analyzed during the current study are not publicly available since they were generated from proprietary data.

## Abbreviations

SBIRT: Screening, Brief Intervention and Referral for Treatment
SUD sample: Substance Use Disorder sample
SUC sample: Substance Use Condition sample
MAT: Medically Assisted Treatment
ICD-10: International Classification of Diseases, 10th Edition
MH condition: Mental Health Condition
